# Clinicopathological and immunological profiles of prostate adenocarcinoma and neuroendocrine prostate cancer

**DOI:** 10.1186/s12957-022-02841-6

**Published:** 2022-12-27

**Authors:** Gang Huang, Huaru Zhang, Haoqing Shi, Wenhui Zhang, Tao Wang, Ziwei Wang, Qing Chen, Bijun Lian, Jing Li, Guosheng Yang

**Affiliations:** 1 Department of Urology, Shanghai East Hospital, Tongji University School of Medicine, Shanghai, 200120 China; 2 Department of Urology, Changhai Hospital, Second Military Medical University, Shanghai, China; 3 Department of Urology, the First Affiliated Hospital of Zhengzhou University, Zhengzhou, Henan China; 4 Department of Urology, the 903th PLA Hospital, Hangzhou, Zhejiang China; 5 Department of Bioinformatics, Center for Translational Medicine, Second Military Medical University, Shanghai, 200433 China

**Keywords:** Adeno-neuroendocrine prostate cancer, Biomarker, Clinicopathological manifestation, Diagnosis, Immunohistochemistry

## Abstract

**Background:**

Biomarkers of DNA damage repair deficiency provide opportunities for personalized treatment with immunotherapy. However, there is limited research on the immune microenvironment of adeno-neuroendocrine prostate cancer (NEPC). In this study, we aimed to assess and describe the comprehensive clinicopathological manifestations of NEPC to improve diagnosis and predict prognosis.

**Methods:**

A retrospective medical record review of 66 patients with prostate cancer (PCa) was performed. PCa samples from the 66 patients were analyzed using immunohistochemical staining for the detection of chromogranin, neural cell adhesion molecule 1, and synaptophysin. For tumor-associated immune microenvironment analysis, PD-L1, CD3, and CD8 were labeled in tissue slides. The effect of clinicopathological factors on the survival of patients with Adeno-NEPC was analyzed.

**Results:**

Twenty patients presented with adeno-NEPC, whereas 46 presented with adeno-PCa. The median age of patients at PCa diagnosis was 67.86 ± 7.05 years (68.65 ± 7.23 years, adeno-NEPC; 67.52 ± 7.02 years, adeno-PCa). Eleven patients with adeno-NEPC underwent prostatectomy, whereas nine received primary androgen deprivation therapy (ADT). Additionally, 30 patients with adeno-PCa underwent prostatectomy, whereas 16 (34.8%) received primary ADT. There was a significant difference in overall survival between patients with adeno-NEPC and those with adeno-PCa (46.0 months vs. 65.0 months). There was also a significant difference in time from prostatectomy to biochemical recurrence between the groups of patients who underwent prostatectomy. Prostatectomy and normal lactate dehydrogenase levels were clinical factors that were significantly associated with better outcomes in patients with adeno-NEPC. Metastatic adeno-NEPC was associated with a higher programmed death ligand 1 (PD-L1) score (2–4) than localized PCa. The data showed that PD-L1 expression in adeno-NEPC may be negatively associated with that in CD8^+^ T cells.

**Conclusions:**

Our study revealed clinicopathological manifestations of adeno-NEPC and some possible predictive factors significantly associated with better outcomes in patients with adeno-NEPC. These findings might be beneficial in the development of diagnostic strategies and customized treatment plans.

**Supplementary Information:**

The online version contains supplementary material available at 10.1186/s12957-022-02841-6.

## Background

Prostate cancer (PCa) is the most commonly diagnosed cancer in men in developed countries and the second leading cause of cancer-related deaths [1]. Adenocarcinoma is the most common pathological type of PCa (> 90%), whereas neuroendocrine PCa (NEPC) is rare at initial diagnosis (approximately 1%) [2]. However, with long-term androgen suppression, approximately one-fifth of patients with castration-resistant PCa (CRPC) present with histologic transformation of the neuroendocrine phenotype, which occurs in relation to the treatment and acts as a castration-resistance mechanism [3, 4]. From diagnosis to death, the survival time of patients with NEPC is usually just 7 months [5]. Thus, there is an urgent need to develop diagnostic strategies and provide more treatment options.

The National Comprehensive Cancer Network guidelines recommend that metastatic tissues from patients with CRPC should be biopsied to confirm NEPC transformation [6]. NEPC can be divided into four pathological subtypes: adenocarcinoma with neuroendocrine differentiation, well-differentiated neuroendocrine tumor, small cell neuroendocrine carcinoma, and large cell neuroendocrine carcinoma [7]. According to previous studies, common neuroendocrine markers include chromogranin (CgA), synaptophysin (SYN), neural cell adhesion molecule 1 (CD56), and neuron-specific enolase (NSE) [8], which are often used to facilitate diagnosis. However, the expression of NE markers is inconsistent because of the heterogeneity of NEPC. It has been reported that NEPC may express one or more NE markers in a sample with morphological changes [9, 10]. Moreover, the detection rate is limited by the complexity of the pathological type. Therefore, the proportion of these NE biomarkers in Chinese patients should be determined.

Currently, there is no effective treatment for NEPC. Patients with adeno-NEPC often receive androgen deprivation therapy (ADT) and chemotherapy (platinum/etoposide) as the first-line treatment; however, prognosis is very poor [11]. Moreover, only a few clinical trials on NEPC treatments have been performed, including cancer stem cell inhibitors (disulfiram [Antabus] and rovalpituzumab tesirine [Rova-T]), epigenetic modulators/EZH2 inhibitors (GSK343 and GSK503), and MYCN and Aurora kinase A inhibitors (e.g., CD532) [11, 12]. Furthermore, biomarkers of DNA damage repair (DDR) deficiency (e.g., BRCA1/2 mutation) provide new opportunities for personalized treatment with immunotherapy (programmed death protein 1 [PD-1]/programmed death ligand 1 [PD-L1] inhibitor) [13]. However, there is limited research on the immune microenvironment of NEPCs.

In the present study, we collected PCa samples from 66 Chinese patients for analysis. Among them, 20 patients had adeno-NEPC, whereas 46 had adeno-PCa. The distribution and expression of common NE biomarkers (CgA, CD56, SYN, and NSE) in the samples were determined using immunohistochemical (IHC) analyses, and some possible predictive factors significantly associated with better outcomes in patients with Adeno-NEPC were assessed. Tumor-infiltrating lymphocytes in the tumor microenvironment were also analyzed to provide a basis for the identification of potential biomarkers for future immunotherapy.

## Methods

### Patients

Patients with PCa who had undergone prostate needle biopsy to confirm the pathologic characteristics of adeno-NEPC and adeno-PCa were retrospectively identified at Changhai Hospital between 2012 and 2019. PCa samples obtained from core needle biopsies were formalin fixed/paraffin embedded. The specimens were evaluated by two experienced pathologists blinded to the clinical characteristics for the determination of consensus pathologic subclassification into two categories (adeno-NEPC and adeno-PCa). Adeno-NEPC was defined by the presence of mixed histology with both adenocarcinoma and neuroendocrine carcinoma using published criteria [8].

The diagnosis of adeno-NEPC was based on morphologic criteria and IHC staining; therefore, IHC analyses were performed for CgA, CD56, SYN, and NSE (Additional file 1: Supplementary Fig. S1). Patients with adeno-NEPC and adeno-PCa were further classified according to different treatments (prostatectomy and ADT) they had received. All analyses were performed in compliance with the Helsinki Declaration of 1975, as revised in 1983, and Good Clinical Practice guidelines. The study protocol was approved by the Changhai Institutional Review Board (CHEC2019-012) (Table 1).

**Table 1:** Clinicopathological characteristics of patients with primary PCa

Characteristic	All *n* = 66	Adeno-NEPC *n* = 20	Adeno-PCa *n* = 46	*P*
Age (years)	67.86 ± 7.05	68.65 ± 7.23	67.52 ± 7.02	0.837
Prostatectomy				0.308
No	27 (40.9%)	11 (55.0%)	16 (34.8%)	
Yes	39 (59.1%)	9 (45.0%)	30 (65.2%)	
Primary ADT				0.308
No	39 (59.1%)	9 (45.0%)	30 (65.2%)	
Yes	27 (40.9%)	11 (55.0%)	16 (34.8%)	
T staging				0.982
T1	1 (1.5%)	0 (0.00%)	1 (2.2%)	
T2	13 (19.7%)	3 (15.0%)	10 (21.7%)	
T3	41 (62.1%)	14 (70.0%)	27 (58.7%)	
T4	11 (16.7%)	3 (15.0%)	8 (17.4%)	
N staging				0.490
N0	37 (56.1%)	9 (45.0%)	28 (60.9%)	
N1	29 (43.9%)	11 (55.0%)	18 (39.1%)	
M staging				0.751
M0	44 (66.7%)	12 (60.0%)	32 (69.6%)	
M1	22 (33.3%)	8 (40.0%)	14 (30.4%)	
Gleason score				0.168
< 7	1 (1.5%)	0 (0.00%)	1 (2.2%)	
7	24 (36.4%)	3 (15.0%)	21 (45.6%)	
> 7	41 (62.1%)	17 (85.0%)	24 (52.2%)	
PSA (ng/L)				0.177
< 10	9 (13.6%)	1 (5.0%)	8 (17.4%)	
10–20	12 (18.2%)	1 (5.0%)	11 (23.9%)	
> 20	45 (68.2%)	18 (90.0%)	27 (58.7%)	
LDH (U/L)				0.207
< 250	57 (86.4%)	15 (75.0%)	42 (91.3%)	
≥ 250	9 (13.6%)	5 (25.0%)	4 (8.7%)	
ALP (U/L)				0.894
< 125	58 (87.9%)	17 (85.0%)	41 (89.1%)	
≥ 125	8 (12.1%)	3 (15.0%)	5 (10.9%)	

*Abbreviations*: *PCa* Prostate cancer, *NEPC* Neuroendocrine prostate cancer, *ADT* Androgen deprivation therapy, *PSA* Prostate-specific antigen, *LDH* Lactate dehydrogenase, *ALP* Alkaline phosphatase

### IHC analysis

A rabbit monoclonal [73-10] PD-L1 antibody (ab228415; Cambridge, UK) was used in the IHC analysis. For tumor-associated lymphocytes, we labeled tissue slides for CD3 and CD8 to highlight the lymphocyte subsets (cytotoxic T cells). The standard IHC PD-L1 score for adeno-NEPC is presented in Additional file 2: Supplementary Fig. S2.

### Outcomes

The primary aim of the study was to evaluate overall survival (OS), which was calculated from the time of initial PCa diagnosis to death. The data of patients who were alive at the time of the last follow-up were censored.

### Statistical analysis

Clinical characteristics, including laboratory values, at the time of biopsy confirming adeno-NEPC and adeno-PCa have been reported. Kaplan–Meier estimates and log-rank tests were used to compare the characteristics of adeno-NEPC with those of adeno-PCa.

## Results

### Clinical characteristics of adeno-NEPC and adeno-PCa

**Fig. 1:**
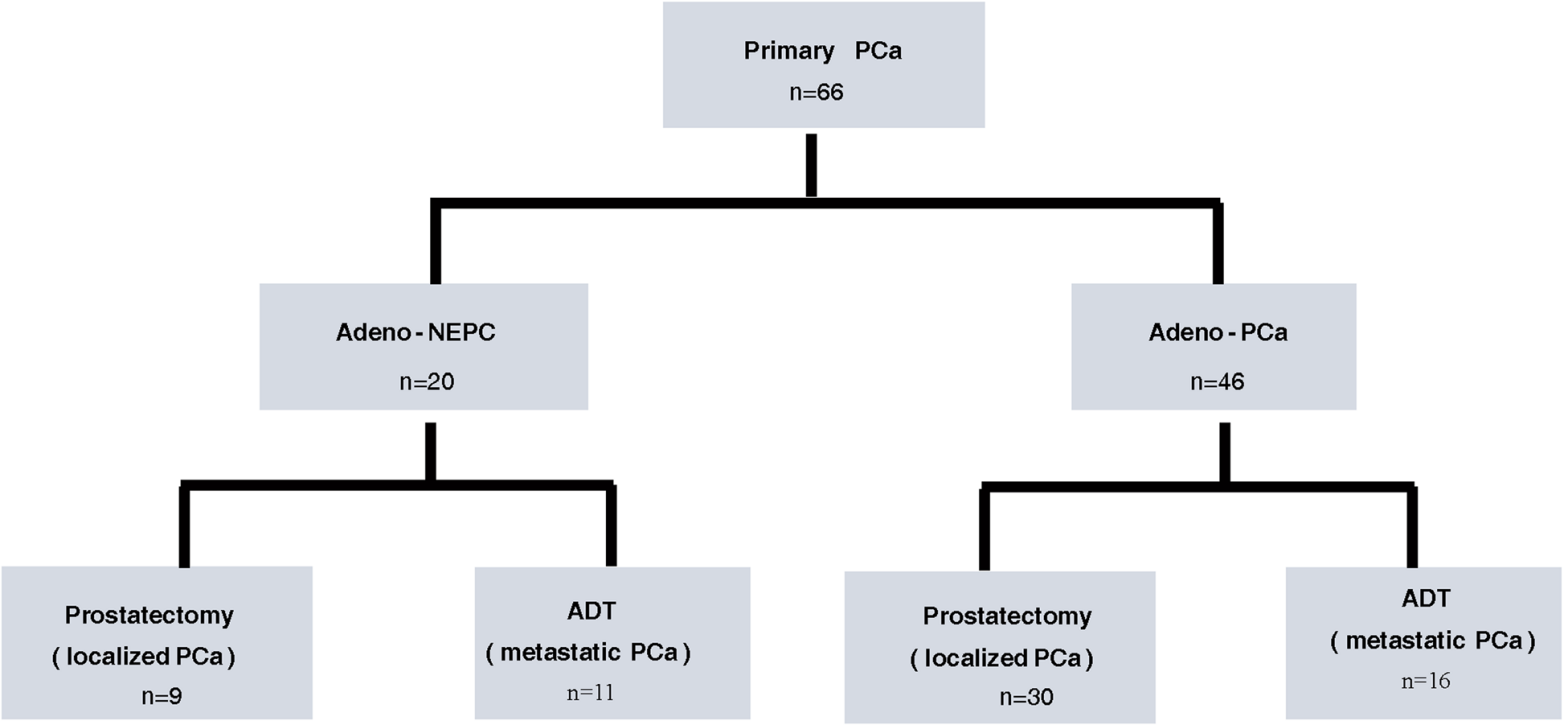
Flow chart of evaluable clinical profiling in patients with primary PCa. PCa, prostate cancer; NEPC, neuroendocrine prostate cancer; ADT, androgen deprivation therapy

Figure 1 shows the two groups into which patients with adeno-NEPC and those with adeno-PCa were further divided according to their initial treatments (prostatectomy and ADT).

The median age at PCa diagnosis was 67.86 ± 7.05 years: 68.65 ± 7.23 years for adeno-NEPC and 67.52 ± 7.02 years for adeno-PCa (*P* = 0.837). Nine (45.0%) patients with adeno-NEPC underwent prostatectomy, whereas 11 (55.0%) received primary ADT. Among patients with adeno-PCa, 30 (65.2%) underwent prostatectomy, whereas 16 (34.8%) received primary ADT. Seventeen (85.0%) patients with adeno-NEPC and 35 (76.1%) with adeno-PCa presented with T3 and T4 at the time of diagnosis. Eleven (55.0%) patients with adeno-NEPC and 18 (39.1%) with adeno-PCa presented with pelvic lymph node involvement at the time of diagnosis. Eight (40.0%) patients with adeno-NEPC and 14 (30.4%) with adeno-PCa presented with metastases at the time of diagnosis. Seventeen (85.0%) patients with adeno-NEPC and 24 (52.2%) with adeno-PCa presented with high-grade Gleason scores (> 7) for their primary tumors. Eighteen (90.0%) patients with adeno-NEPC and 27 (58.7%) with adeno-PCa had high prostate-specific antigen (PSA) levels > 20 ng/L) at the time of diagnosis. Five (25.0%) patients with adeno-NEPC and four (8.7%) with adeno-PCa had lactate dehydrogenase (LDH) levels ≥ 250 U/L at the time of diagnosis. Three (15.0%) patients with adeno-NEPC and five (10.9%) with adeno-PCa had alkaline phosphatase levels ≥ 125 U/L at the time of diagnosis. Detailed clinicopathological manifestations of patients with adeno-NEPC and adeno-PCa at the time of diagnosis are summarized in the following:

### Heat map of NE biomarkers in adeno-NEPC

**Fig. 2:**
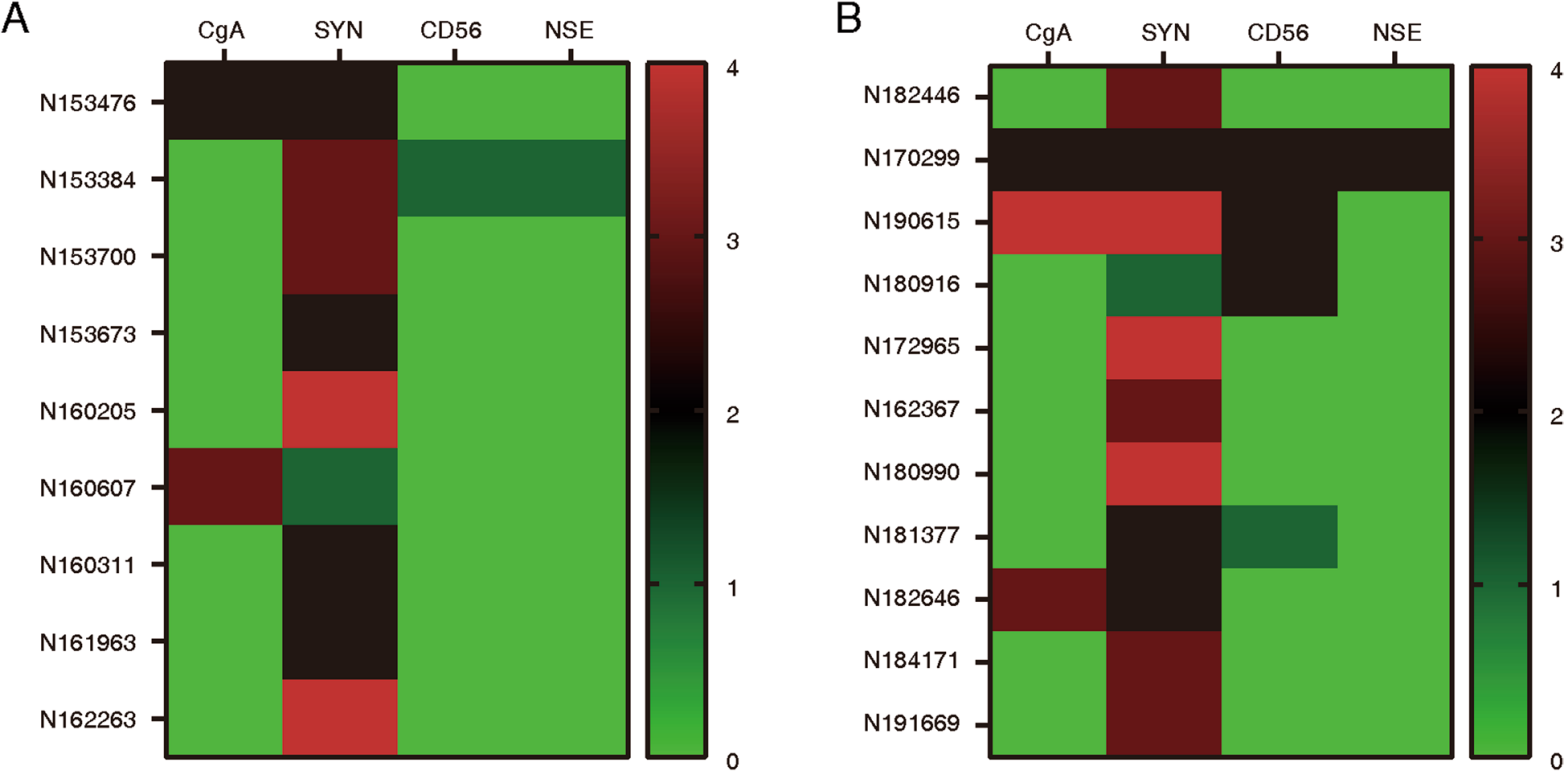
Heat map of NE biomarkers in adeno-NEPC. **a** Heat map of NE biomarkers in localized adeno-NEPC. **b** Heat map of NE biomarkers in metastatic adeno-NEPC. CgA, chromogranin; SYN, synaptophysin; CD56, neural cell adhesion molecule 1; NSE, neuron-specific enolase

Adeno-NEPC samples from 20 patients were stained for the analysis of NE biomarkers (SYN, CgA, CD56, and NSE). Co-expression of CgA, SYN, CD56, and NSE in adeno-NEPC is demonstrated in Additional file 3: Supplementary Fig. S3. We used a heat map to describe the distribution and expression of NE biomarkers in each sample (Fig. 2). The expression ratios of SYN, CgA, CD56, and NSE in the samples were 100%, 25%, 25%, and 10%, respectively.

### Comparison of the clinical characteristics of adeno-NEPC

**Fig. 3:**
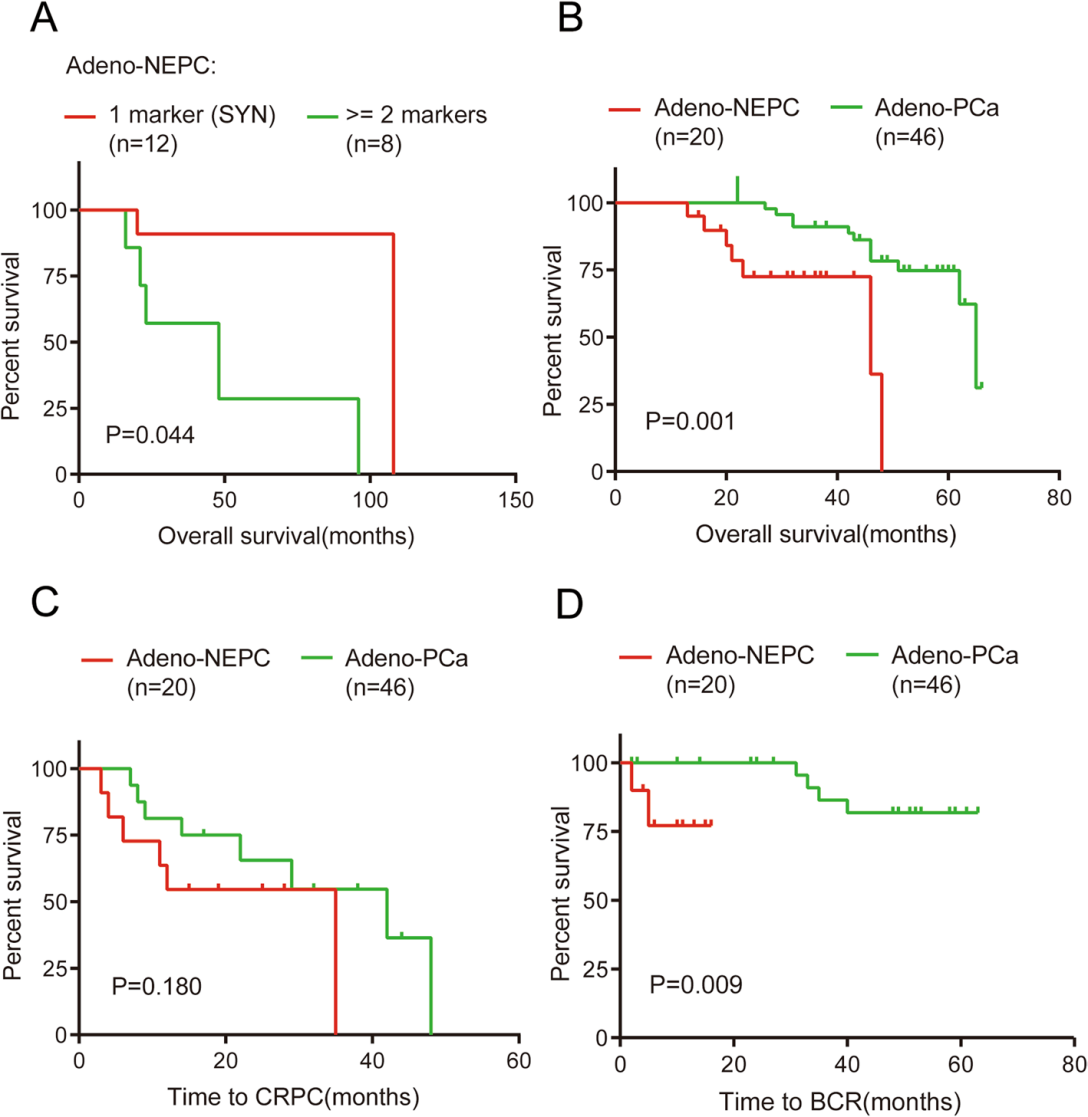
Kaplan–Meier survival curves basing on NE biomarker expression and pathological subtype. **a** OS analyses between adeno-NEPC samples positive for only SYN and those positive for more than one NE biomarker. **b** OS analyses between adeno-NEPC and adeno-PCa. **c** Time to CRPC analyses between adeno-NEPC and adeno-PCa. **d** Time to BCR analyses between adeno-NEPC and adeno-PCa. PCa, prostate cancer; NEPC, neuroendocrine prostate cancer; SYN, synaptophysin; CRPC, castration-resistant prostate cancer; BCR, biochemical recurrence

The adeno-NEPC samples were divided into two groups based on the expression of NE markers. Twelve of the 20 adeno-NEPC samples were positive for only SYN, whereas the remaining samples were positive for more than one NE biomarker. The clinical characteristics of the two groups are compared in Table 2. There was a significant difference in OS between the groups (Fig. 3a).

**Table 2:** Clinicopathological characteristics of patients with adeno-NEPC

Characteristic	Only SYN (+) *n* = 12	Not only SYN (+) *n* = 8	*P*
Age (years)	66.00 ± 2.00	72.63 ± 2.12	0.041
T staging			0.528
T2	3 (25.0%)	1 (12.5%)	
T3	8 (66.7%)	5 (62.5%)	
T4	1 (8.3%)	2 (25.0%)	
N staging			0.142
N0	7 (58.3%)	2 (25.0%)	
N1	5 (41.7%)	6 (75.0%)	
M staging			0.852
M0	7 (58.3%)	5 (62.5%)	
M1	5 (41.7%)	3 (37.5%)	
Gleason score			0.798
≤ 7	2 (16.7%)	1 (12.5%)	
> 7	10 (83.3%)	7 (87.5%)	
PSA (ng/L)			0.224
≤ 20	2 (16.7%)	0 (0.0%)	
> 20	10 (83.3%)	8 (100.0%)	
LDH (U/L)			0.999
< 250	9 (75.0%)	6 (75.0%)	
≥ 250	3 (25.0%)	2 (25.0%)	
ALP (U/L)			0.798
< 125	10 (83.3%)	7 (87.5%)	
≥ 125	2 (16.7%)	1 (12.5%)	

*Abbreviations*: *SYN* Synaptophysin, *PSA* Prostate-specific antigen, *LDH* Lactate dehydrogenase, *ALP* Alkaline phosphatase

### *OS analyses*

There was a significant difference in OS between patients with adeno-NEPC and those with adeno-PCa (median OS from PCa diagnosis: 46.0 months vs 65.0 months, *P* = 0.001). In the cohort of patients who received ADT, the time from the diagnosis of hormone-sensitive PCa (HSPC) to that of CRPC was not significantly different between the groups (*P* = 0.18). However, in the cohort of patients who underwent prostatectomy, we observed a significant difference in the time from prostatectomy to biochemical recurrence (BCR) between the groups (*P* = 0.0086) (Fig. 3b–d).

### Univariate Cox regression analysis for OS in patients with adeno-NEPC and response to different treatments

Prostatectomy and normal LDH levels were clinical factors significantly associated with better outcomes in patients with adeno-NEPC (Table 3).

**Table 3:** Univariate Cox regression analysis for overall survival in patients with adeno-NEPC

Variable	Hazard ratio (95% *CI*)	*P*
Prostatectomy (yes vs. no)	0.177 (0.036–0.883)	0.013
T staging (< T3b vs. ≥ T3b)	1.420 (0.299–6.739)	0.237
N staging (N0 vs. N1)	0.467 (0.106–2.066)	0.311
M staging (M0 vs. M1)	0.315 (0.048–2.073)	0.086
Gleason score (≤ 8 vs. > 8)	0.368 (0.074–1.822)	0.283
LDH (< 250 vs. ≥ 250)	0.195 (0.031–1.218)	0.011

*Abbreviations*: *LDH* Lactate dehydrogenase

Nine of the 20 patients with adeno-NEPC (45.0%) underwent prostatectomy. Subsequently, 6 (30.0%) received ADT, 5 (25.0%) underwent radiotherapy, 2 (10.0%) received chemotherapy, and 2 (10.0%) received abiraterone (median OS = 48.0 months). Additionally, among the 20 patients, 11 (55.0%) received primary ADT, and subsequently, 5 (25.0%) received chemotherapy, and 4 (20.0%) received abiraterone or enzalutamide (median OS = 21.0 months).

### PD-L1 expression in adeno-NEPC and its possible negative correlation with CD8^+^ T-cell expression

**Fig. 4:**
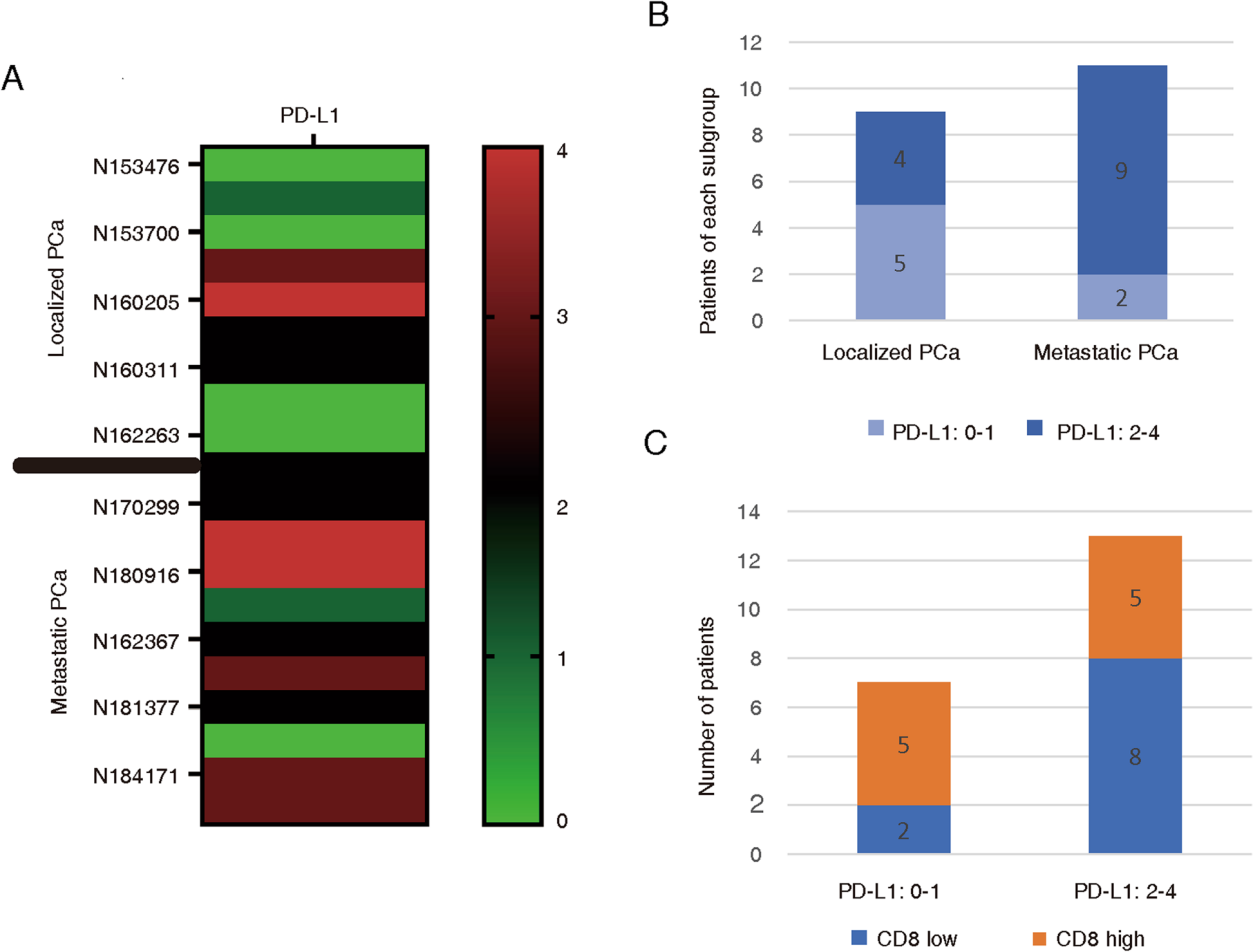
PD-L1 expression in adeno-NEPC and its possible negative correlation with CD8+ T-cell expression. **a** Heat map used to describe the distribution and expression of PD-L1 in each adeno-NEPC sample. **b** Histogram used to demonstrate a trend toward a higher PD-L1 score (2–4) in metastatic PCa. **c** Histogram used to demonstrate a trend toward a lower expression of CD8^+^ T cells in patients with higher PD-L1 scores (2–4). PD-L1, programmed death-ligand 1

Samples from the 20 patients with adeno-NEPC were stained for PD-L1 analysis. The positive expression rate of PD-L1 in these cases was 75% (15/20). A heat map was used to describe the distribution and expression of PD-L1 in each sample (Fig. 4a). The patients were divided into two groups according to their clinical stages. Metastatic PCa demonstrated a trend toward a higher PD-L1 score (2–4) than localized PCa (Fig. 4b). In addition, patients with higher PD-L1 scores (2–4) demonstrated a trend toward lower expression of CD8^+^ T cells, whereas those with lower PD-L1 scores (0–1) demonstrated a trend toward higher expression of CD8^+^ T cells (Fig. 4c).

## Discussion

Widespread use of new hormonal treatments such as abiraterone and enzalutamide has substantially increased the incidence of NEPC [14]. Potent androgen blockade prominently promotes neuroendocrine differentiation of prostatic adenocarcinoma, which acts as a mechanism of therapeutic resistance [15]. Given the morphological and pathological similarities between NEPC and small cell lung cancer (SCLC), the current first-line treatment for patients with NEPC is chemotherapy, which includes the use of etoposide plus cisplatin [16]. Combination therapy (continuous ADT and early-stage chemotherapy) is advisable for adeno-NEPC management. We reviewed the clinicopathological characteristics of a cohort of patients with adeno-NEPC and have provided insights into specific clinical variables that might aid in predicting prognosis.

The OS of patients with adeno-NEPC was significantly shorter than that of patients with adeno-PCa. Nevertheless, in the cohort of patients receiving ADT, the difference in the time from HSPC development to CRPC development between the groups was not significant, which partially resulted from the diverse mechanisms of CRPC. Neuroendocrine differentiation is a response to prostatic adenocarcinoma under the pressure of continuous endocrine therapy. The clinical features of NEPC are androgen receptor (AR) deficiency, low PSA level, and frequent loss of *RB1* and *TP53* [17]. The AR-independent growth of NEPC contributes to the development of CRPC. In addition, other factors might be involved in the progression of CRPC, including tumor immunity, cancer stem cells, epithelial-mesenchymal transition (EMT), and DNA repair disorders [18]. As an aggressive subtype of CRPC, NEPC has a worse prognosis. Adeno-NEPC is considered as the transitional stage that presents similar clinical features to those of NEPC.

About one-third of patients develop BCR during the first 10 years after prostatectomy [19]. In the present study, a significant difference in the time from prostatectomy to BCR was observed between the groups of patients who had undergone prostatectomy. Furthermore, patients with higher PSA levels at diagnosis, older age at surgery, and higher pathological grading and staging are more prone to late BCR [20]. The time to BCR is significantly correlated with the time of cancer-specific death [19, 20]. Therefore, it is recommended that patients with adeno-NEPC should be followed up closely after surgery because of the shorter time from prostatectomy to BCR among them.

LDH is a common enzyme in major organs that is usually involved in cell damage, death, inflammation, and hemolysis [21]. Given that a hypoxic microenvironment results in increased lactic acid levels, serum LDH level is correlated with cancer progression, malignant transformation, and angiogenesis [22, 23]. Furthermore, increased serum LDH level is considered an independent prognostic factor for OS in patients with CRPC [24]. In the present study, high levels of LDH (≥ 250 U/L) were associated with worse outcomes in patients with adeno-NEPC, which indicates that LDH is a prognostic biomarker for the aggressive subtype of CRPC.

We focused on adeno-NEPC with dual expression of neuroendocrine markers and AR in the same tumor samples resulting from tumor heterogeneity. Adeno-NEPC contains prostatic adenocarcinoma and neuroendocrine differentiation, which is considered as the transitional stage that presents similar clinical features to those of NEPC. Adeno-NEPC samples in this study showed hybrid characteristics representing AR and neuroendocrine markers simultaneously. So, most adeno-NEPC patients have higher PSA value than NEPC patients have. On the other hand, the mixed status between prostatic adenocarcinoma and NEPC could be characterized by defects in DDR [25]. Tumor mutational burden (TMB) and defects in DDR genes are with the effectiveness of immune checkpoint blockade (ICB) therapies [26]. As observed in other cancers [27, 28], DDR defects could potentially contribute to the immune sensitization of PCa to ICB. Accordingly, a combined therapeutic strategy, including the use of hormonal drugs and other chemotherapeutic agents (platinum/etoposide) or ICB therapies (such as PD-1/PD-L1 inhibitors), is recommended to overcome resistance related to neuroendocrine transformation.

High PD-L1 expression is associated with a poor clinical prognosis of PCa [29]. A previous study reported that PD-L1-positive tumors are four times more likely to metastasize than PD-L1-negative tumors [30]. In the present study, metastatic adeno-NEPC demonstrated a trend toward a higher PD-L1 score (2–4) than localized adeno-NEPC, which indicates that moderate to high PD-L1 expression is possibly related to a poor prognosis of PCa. Furthermore, in localized PCa after radical prostatectomy, both high CD8 expression and low PD-L1 expression were significantly associated with a longer time to BCR, which showed that combined assessment of markers could better predict prognosis [31]. Moreover, the interaction between PD-L1 and PD-1 is an important checkpoint for CD8^+^ T-cell inhibition [32]. In the present study, patients with adeno-NEPC with higher PD-L1 scores (2–4) demonstrated a trend toward lower expression of CD8^+^ T cells, which might contribute to an immunosuppressive microenvironment.

There were several limitations to our study, including the study design (retrospective non-randomized trial), modest number of patients, and lack of molecular features of adeno-NEPC. Despite these limitations, our study provides clinicopathological manifestations of adeno-NEPC and some possible predictive factors significantly associated with better outcomes in patients with adeno-NEPC.

## Conclusions

Our findings show that patients with adeno-NEPC should be followed up closely after surgery, which could help in detecting BCR early for timely treatment. High levels of LDH (≥ 250 U/L) might be a poor prognostic biomarker in patients with adeno-NEPC. Additionally, therapeutic options for adeno-NEPC, such as ICB treatments, are recommended to potentially improve the prognosis of CRPC.

## Supplementary Information

**Additional file 1: Figure S1.** IHC staining for SYN, CgA and CD56 in Adeno-NEPC.

**Additional file 2: Figure S2.** The standard IHC PD L1 scores in Adeno-NEPC.

**Additional file 3: Figure S3.** Co expression of CgA, SYN, CD56 and NSE in Adeno-NEPC.

**Additional file 4: Supplementary Table 1.** Univariate Cox regression analysis for overall survival in patients with Adeno-NEPC.

**Additional file 5: Supplementary Table 2.** Clinicopathological characteristics of patients with Adeno-NEPC.

## Data Availability

The data of this study are available from the corresponding authors on reasonable request.

## Abbreviations

ADT: Androgen deprivation therapy
ALP: Alkaline phosphatase
BCR: Biochemical recurrence
CgA: Chromogranin
CRPC: Castration-resistant prostate cancer
DDR: DNA damage repair
HSPC: Hormone-sensitive prostate cancer
ICB: Immune checkpoint blockade
IHC: Immunohistochemistry
LDH: Lactate dehydrogenase
NEPC: Neuroendocrine prostate cancer
NSE: Neuron-specific enolase
OS: Overall survival
PCa: Prostate cancer
PD-L1: Programmed death ligand 1
PSA: Prostate-specific antigen
SCLC: Small cell lung cancer
SYN: Synaptophysin
TMB: Tumor mutational burden
EMT: Epithelial–mesenchymal transition
