# Supplementary figures and images for: Clinicopathological and immunological profiles of prostate adenocarcinoma and neuroendocrine prostate cancer
Source: World J Surg Oncol. 2022 Dec 27;20:407. doi: 10.1186/s12957-022-02841-6 (PMC9793563; doi:10.1186/s12957-022-02841-6)

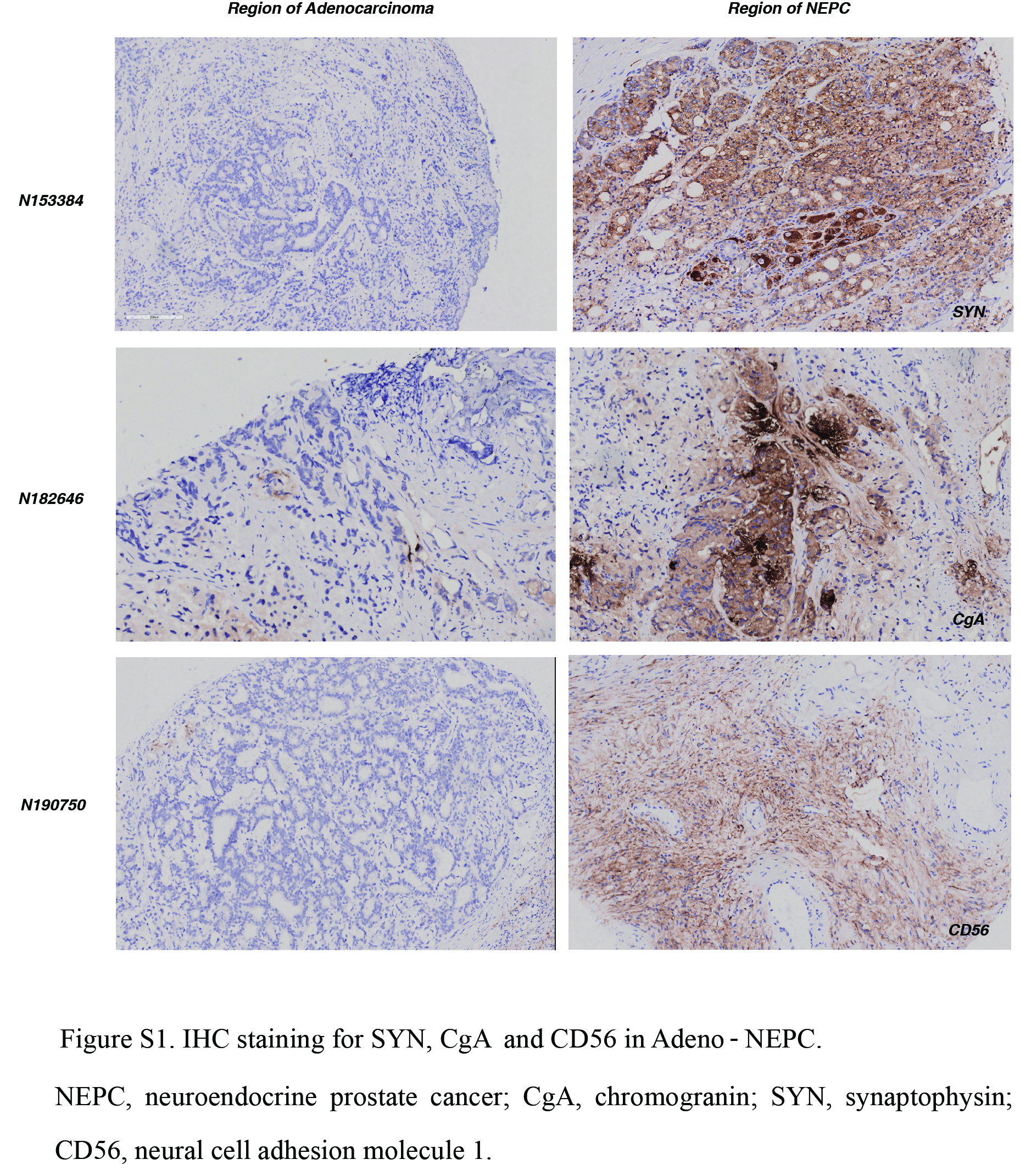

Supplement: Supplementary file 1 — Additional file 1: Figure S1. IHC staining for SYN, CgA and CD56 in Adeno-NEPC. [file 12957_2022_2841_MOESM1_ESM.tif]

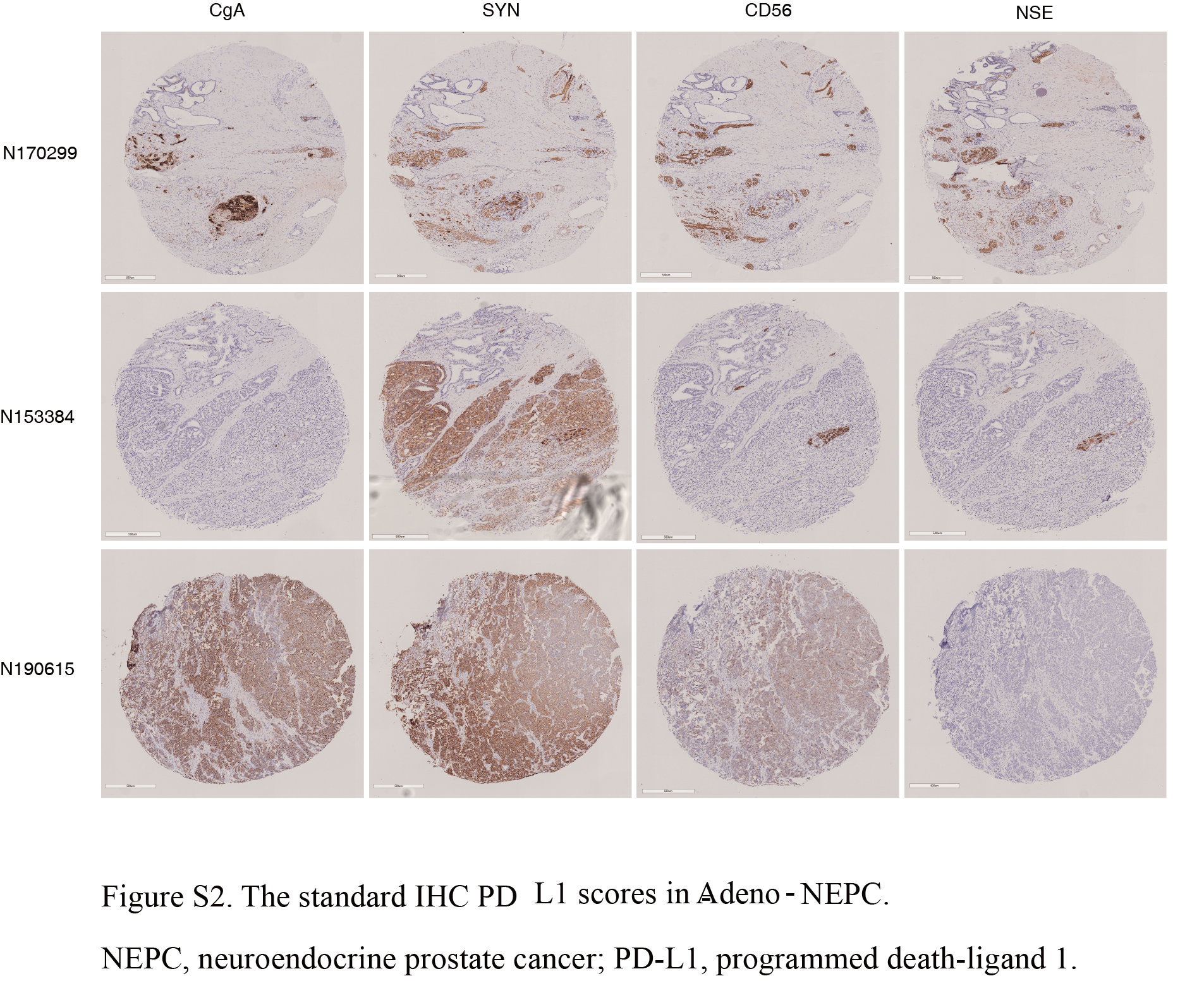

Supplement: Supplementary file 2 — Additional file 2: Figure S2. The standard IHC PD L1 scores in Adeno-NEPC. [file 12957_2022_2841_MOESM2_ESM.tif]

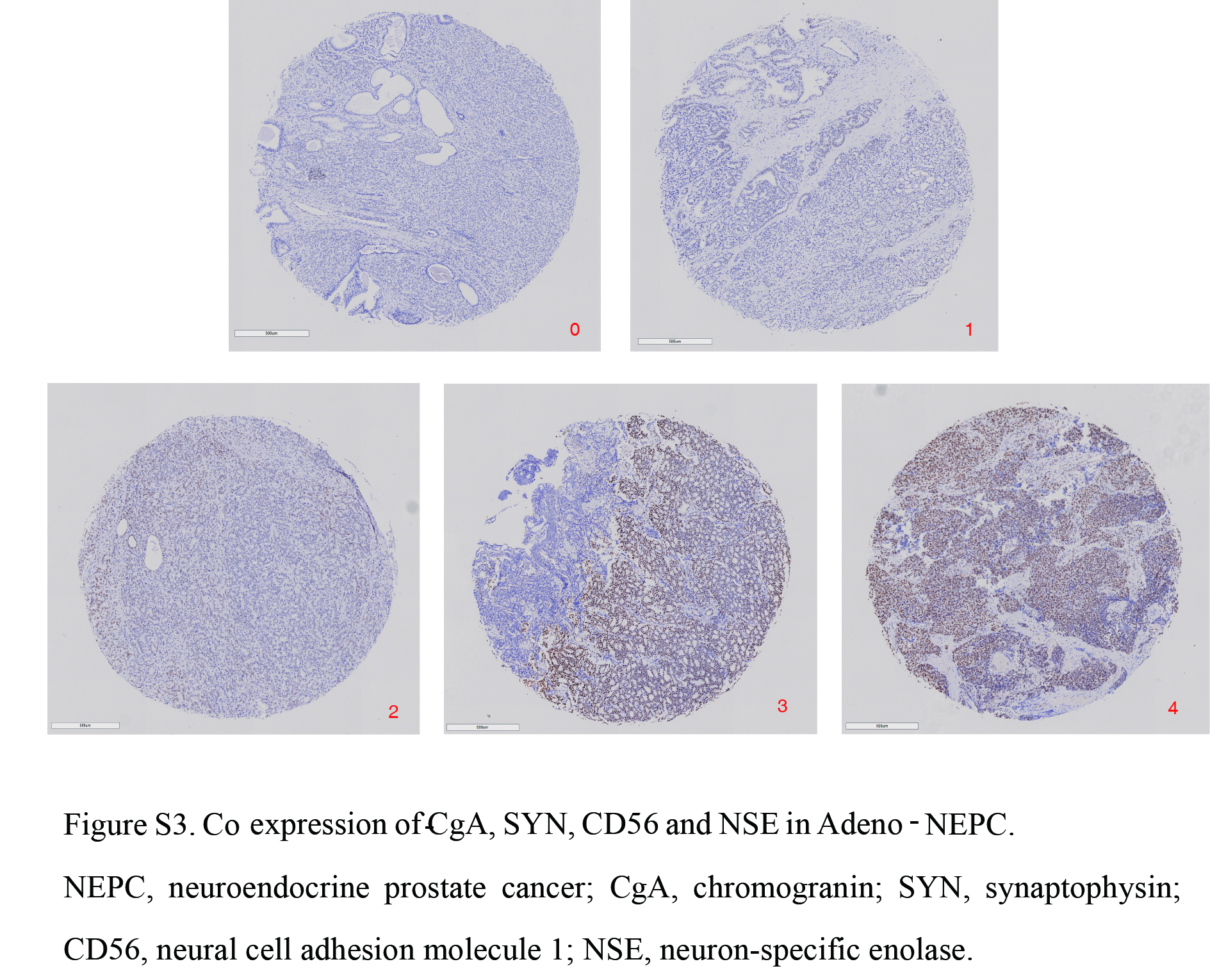

Supplement: Supplementary file 3 — Additional file 3: Figure S3. Co expression of CgA, SYN, CD56 and NSE in Adeno-NEPC. [file 12957_2022_2841_MOESM3_ESM.tif]
