# Supplementary material for: Clinicopathological and immunological profiles of prostate adenocarcinoma and neuroendocrine prostate cancer
Source: World J Surg Oncol. 2022 Dec 27;20:407. doi: 10.1186/s12957-022-02841-6 (PMC9793563; doi:10.1186/s12957-022-02841-6)
Supplement: Supplementary file 4 — Additional file 4: Supplementary Table 1. Univariate Cox regression analysis for overall survival in patients with Adeno-NEPC. [file 12957_2022_2841_MOESM4_ESM.docx]

Supplementary Table 1. Univariate Cox regression analysis for overall survival in patients with Adeno-NEPC

| **Variable** | **Hazard ratio (95% CI)** | ***P* value** |
| --- | --- | --- |
| **Prostatectomy (yes vs. no)** | 0.177[0.036-0.883] | 0.013 |
| **T staging (<T3b vs. ≥T3b)** | 1.420[0.299-6.739] | 0.237 |
| **N staging (N0 vs. N1)** | 0.467[0.106-2.066] | 0.311 |
| **M staging (M0 vs. M1)** | 0.315[0.048-2.073] | 0.086 |
| **Gleason Score (****≤8 vs. >8)** | 0.368[0.074-1.822] | 0.283 |
| **LDH (<250 vs. ≥250)** | 0.195[0.031-1.218] | 0.011 |

Abbreviations: LDH, lactate dehydrogenase.
