# Supplementary material for: Clinicopathological and immunological profiles of prostate adenocarcinoma and neuroendocrine prostate cancer
Source: World J Surg Oncol. 2022 Dec 27;20:407. doi: 10.1186/s12957-022-02841-6 (PMC9793563; doi:10.1186/s12957-022-02841-6)
Supplement: Supplementary file 5 — Additional file 5: Supplementary Table 2. Clinicopathological characteristics of patients with Adeno-NEPC. [file 12957_2022_2841_MOESM5_ESM.docx]

Supplementary Table 2. Clinicopathological characteristics of patients with Adeno-NEPC.

| Characteristics | PD-L1: 0-1  n=7 | PD-L1: 2-4  n=13 | *P* value |
| --- | --- | --- | --- |
| Age(years) | 71.43 ± 2.82 | 67.15 ± 1.92 | 0.216 |
| T staging |  |  | 0.354 |
| T2 | 2(28.6%) | 2(15.4%) |  |
| T3 | 5(71.4%) | 8(61.5%) |  |
| T4 | 0(0.0%) | 3(23.1%) |  |
| N staging |  |  | 0.888 |
| N0 | 3(42.9%) | 6(46.2%) |  |
| N1 | 4(57.1%) | 7(53.8%) |  |
| M staging |  |  | 0.444 |
| M0 | 5(71.4%) | 7(53.8%) |  |
| M1 | 2(28.6%) | 6(46.2%) |  |
| Gleason Score |  |  | 0.212 |
| ≤7 | 2(28.6%) | 1(7.7%) |  |
| >7 | 5(71.4%) | 12(92.3%) |  |
| PSA (ng/L) |  |  | 0.948 |
| ≤20 | 1(16.7%) | 2(15.4%) |  |
| >20 | 6(83.3%) | 11(84.6%) |  |
| LDH(U/L) |  |  | 0.417 |
| <250 | 6(83.3%) | 9(69.2%) |  |
| ≥250 | 1(16.7%) | 4(30.8%) |  |
| ALP(U/L) |  |  | 0.212 |
| <125 | 5(71.4%) | 12(92.3%) |  |
| ≥125 | 2(28.6%) | 1(7.7%) |  |

Abbreviations: PSA, prostate-specific antigen; LDH, lactate dehydrogenase; ALP, alkaline phosphatase.
